# Time series modelling to forecast prehospital EMS demand for diabetic emergencies

**DOI:** 10.1186/s12913-017-2280-6

**Published:** 2017-05-05

**Authors:** Melanie Villani, Arul Earnest, Natalie Nanayakkara, Karen Smith, Barbora de Courten, Sophia Zoungas

**Affiliations:** 1 Monash Centre for Health Research and Implementation – MCHRI, School Public Health and Preventive Medicine, Monash University in partnership with Monash Health, 43 – 51 Kanooka Grove, Clayton, Victoria 3168 Australia; 2 Research and Evaluation, Ambulance Victoria, 375 Manningham Road, Doncaster, Victoria 3108 Australia; 3 Diabetes and Vascular Medicine Unit, Monash Health, 246 Clayton Road, Clayton, Victoria 3168 Australia; 4 Department of Epidemiology and Preventive Medicine, School of Public Health and Preventive Medicine, Monash University, Alfred Hospital, Commercial Road, Victoria, 3004 Australia; 5 Department of Emergency Medicine, School of Primary, Aboriginal and Rural Health Care, University of Western Australia, Crawley, Western Australia 6009 Australia; 6 The George Institute for Global Health, Camperdown, New South Wales 2050 Australia; 7 School of Public Health and Preventive Medicine, Monash University, Locked bag 29, Monash Medical Centre, Clayton, Victoria 3168 Australia

**Keywords:** Access/Demand/Utilization of services, Diabetes, Emergency medical services, Time series analysis

## Abstract

**Background:**

Acute diabetic emergencies are often managed by prehospital Emergency Medical Services (EMS). The projected growth in prevalence of diabetes is likely to result in rising demand for prehospital EMS that are already under pressure. The aims of this study were to model the temporal trends and provide forecasts of prehospital attendances for diabetic emergencies.

**Methods:**

A time series analysis on monthly cases of hypoglycemia and hyperglycemia was conducted using data from the Ambulance Victoria (AV) electronic database between 2009 and 2015. Using the seasonal autoregressive integrated moving average (SARIMA) modelling process, different models were evaluated. The most parsimonious model with the highest accuracy was selected.

**Results:**

Forty-one thousand four hundred fifty-four prehospital diabetic emergencies were attended over a seven-year period with an increase in the annual median monthly caseload between 2009 (484.5) and 2015 (549.5). Hypoglycemia (70%) and people with type 1 diabetes (48%) accounted for most attendances. The SARIMA (0,1,0,12) model provided the best fit, with a MAPE of 4.2% and predicts a monthly caseload of approximately 740 by the end of 2017.

**Conclusions:**

Prehospital EMS demand for diabetic emergencies is increasing. SARIMA time series models are a valuable tool to allow forecasting of future caseload with high accuracy and predict increasing cases of prehospital diabetic emergencies into the future. The model generated by this study may be used by service providers to allow appropriate planning and resource allocation of EMS for diabetic emergencies.

## Background

The prevalence of diabetes is increasing [1] and is becoming one of the most significant health issues of the developed world [2]. In Australia 1.24 million people are registered as having diabetes [3] and an additional 500,000 are estimated to have undiagnosed diabetes [4]. Between 2013 and 2016, this number increased by 4.3% per year with the increase in the prevalence of type 2 diabetes greater (4.4% increase per year) than that of type 1 diabetes (0.6% increase per year) [3]. The impact of diabetes on the health system is widespread and includes both acute and chronic complications. Patients experiencing acute glycemic complications, most frequently severe hypoglycemia and hyperglycemic crises (diabetic ketoacidosis and hyperglycemic hyperosmolar state), will often seek emergency medical assistance in the community from the prehospital Emergency Medical Services (EMS) [5]. When combined with the rising demand for prehospital EMS [6] [7], the increasing diabetes prevalence will require careful resource planning.

Health forecasting, the prediction of health or disease episodes and signaling of future events, is increasingly recognized as a valuable tool to facilitate health service provision and resource allocation. Health forecasting is often based on a time series, a sequence of data points collected at successive, equally spaced time intervals, which may be characterised by trend, seasonality, cyclicality and randomness [8]. A time series provides a statistical means of temporal trend analysis and prediction of future events based on observed values. There are various forecasting methods in the health literature, including historical averaging [9], smoothing techniques [10, 11], linear regression [12] and autoregressive integrated moving average (ARIMA) modelling [13, 14]. ARIMA modelling has demonstrated successful prediction of a range of specific health disease events [15, 16] as well as in aggregate caseload, such as hospital emergency department [9–11, 13] and prehospital EMS [17] and has been recognised for its simplicity and ease of administration [18].

Temporal trends in diabetic emergencies have been investigated at the hospital level [19] [20] but trends of demand for prehospital EMS for acute diabetic emergencies have not been reported. The aims of this study were 1) to quantify the temporal trends in utilization of prehospital EMS for acute diabetic emergencies, 2) to model the temporal variation of prehospital diabetic emergencies and 3) to use the model to make short term predictions of future EMS demand for diabetic emergencies.

## Methods

A time series analysis on monthly case rates of diabetic emergencies attended by Ambulance Victoria (AV) between January 2009 and December 2015 was conducted. AV is a two-tiered, prehospital EMS system and the sole provider of prehospital EMS for the state of Victoria, Australia. Individuals of all ages receiving prehospital care from AV during the study period with a documented final primary assessment of “hyperglycemia” or “hypoglycemia” were included. The final primary assessment, as assigned by the attending paramedic, is defined as the main problem at the time the patient is discharged from EMS care. No blood glucose level threshold parameters were imposed, however AV uses a BGL < 4 mmol/L (<72 mg/dl) to treat for hypoglycemia and does not specify a glycemic threshold for hyperglycemia. Every case attended by AV is recorded by the attending paramedic, using the VACIS®, an electronic patient care record and integrated data warehouse [21]. Diabetes type, based on patient/bystander self-report, was classified as type 1 diabetes, type 2 diabetes or unspecified diabetes type/status. In this study de-identified data was used with no ability to distinguish repeat callers, thus repeat attendances were treated as individual cases. Data spanning 4 calendar months (September 2014 – December 2014) were unavailable due to lapse in electronic data collection linked to industrial action. The Monash Health Human Research Ethics Committee approved this study.

Descriptive analysis was conducted by tabulating total annual case count and median monthly case count for each year. Categorical variables (gender, diabetes type, emergency type) are reported as absolute number and percentage, and differences between subgroups analysed using *χ*2 test. Age was summarised as median with interquartile range (IQR), and differences between subgroups analysed using Kruskal-Wallis test.

The autoregressive integrated moving average (ARIMA) modelling process was conducted using monthly case counts during the study period, initially with total case count and then separately for hypoglycemia/hyperglycemia and for male/female. ARIMA models make use of previous observations to make predictions of future values using lag parameter values, under the assumption that the pattern will persist. Lags of the differenced series, termed Auto Regressive (AR), indicate the strength of relationship between incidence rates in consecutive months and lags of the forecast errors, termed Moving Average (MA), check for dependence of monthly incidence on current and past model residuals. A differencing term, D, is applied to make the data stationary when the time series displays a long term trend and a seasonal term, S, is incorporated when the time series displays a seasonal pattern, and Seasonal ARIMA (SARIMA) modelling is used.

The data was divided into two sets; training data and validation data. This was because models are expected to perform well on the data from which they were derived, so post sample validation on a prospective ‘new’ dataset was performed. Model *generation* was based on the dataset; January 2009 - August 2014 (training dataset) and model *validation* was based on the dataset; January 2015 - December 2015 (validation dataset). The Box-Jenkins approach was used to fit the models [22]. Annual seasonality was apparent in the plotted monthly caseload (Fig. 2), thus SARIMA modelling with a 12-month seasonality term was used. The Autocorrelation Function (ACF) and Partial Autocorrelation Function (PACF) were plotted to examine stationarity and lags and to assist in identification of the order the MA and AR terms of each model.

Different formulations of the AR and MA terms were modelled. The following measures of prediction accuracy were calculated; Mean Absolute Error (MAE), Mean Square Error (MSE) and Mean Absolute Percentage Error (MAPE), defined as:

$$ \begin{array}{l}\mathbf{MAE} = \frac{1}{n}{\displaystyle \sum_{i=1}^n}\left| Oi- Pi\right|\\ {}\mathbf{MSE}\kern0.5em =\kern0.5em \frac{1}{n}{\displaystyle \sum_{i=1}^n}{\left( Oi- Pi\right)}^2\\ {}\mathbf{MAPE} = \kern0.5em \frac{100}{n}\ {\displaystyle \sum_{i=1}^n}\left|\frac{Oi- Pi}{Oi}\right|\end{array} $$

where *Oi*, *Pi* and *n* are the observed and predicted counts for month “*i*” and the number of observations, respectively and where a lower prediction error indicates a better model fit. Each model was used to predict values for the training dataset and the validation dataset. The most parsimonious model with lower MAE, MSE and MAPE values for each condition (overall caseload, hypoglycaemia, hyperglycaemia, female and male) was selected. The final selected model for each condition was then used to generate predicted monthly caseload values until the December 2017. The predicted values for the training data were based on a one-step forecast while the predicted values for the validation dataset and the 2017 forecast were based on dynamic forecasting, commencing on August 2014, the final month of training dataset. Compared to one-step forecasting, which bases forecasts on *observed* values of the preceding month, dynamic forecasts are based on *predicted* values of the preceding month, thus are susceptible to errors accumulating over time.

The chosen SARIMA model for overall caseload was then used to generate 1-month forecasts, 3-month forecasts and 12-month forecasts, and the MAE, MSE and MAPE were calculated for each, using dynamic forecast predictions. In addition, the chosen SARIMA model was compared to other modelling techniques for overall caseload. The models generated for comparison were; a) SARIMA, b) SARIMA + time trend, c) ARIMA + seasonality, d) exponential smoothing and e) linear time trend + seasonality. Modelling was performed using one-step forecasts and model accuracy was compared using the three accuracy measures; MAE, MSE and MAPE. All analyses were performed using Stata software version 14.0 (StataCorp, Texas, USA) and the level of significance was set at 5%.

## Results

**Fig. 1:**
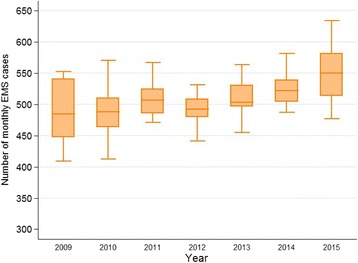
Median monthly EMS attendance for diabetic emergencies

During the 7-year study period 41,454 prehospital diabetic emergencies were attended by AV, equating to an annual mean (±SD) of 5922 ± 508 cases. Table 1 displays the total annual case load for each year, median age and the proportional distribution of gender, type of diabetes and type of emergency. The median [IQR] monthly attendance over the study period was 506 [481, 534], with an increasing trend in median monthly attendance per year between 2009 (484.5 [447.5, 540.5]) and 2015 (549.5 [513.5, 581.5]) (Fig. 1). Gender distribution (approximately 55% male) remained relatively stable and median [IQR] age decreased very slightly from 60 [40, 76] years to 59 [39, 75] years over the study period (*p* = 0.11). Overall, hypoglycemia accounted for 70% of attendances however, over the study period, the proportion of attendances for hypoglycemia decreased (75% to 63%) and the proportion of hyperglycemia increased (25 to 37%) (*p* < 0.001). Attendances to people with type 1 diabetes and type 2 diabetes accounted for 48% and 39% of overall attendances, respectively. The relative proportion of attendances to persons with type 1 diabetes and type 2 diabetes decreased (53% to 44%) and increased (35 to 42%), respectively (*p* < 0.001).

**Table 1:** Descriptive characteristics of prehospital diabetic emergencies

Year	2009	2010	2011	2012	2013	2014	2015	*p*-value
Annual cases (n)	5841	5873	6102	5911	6179	4938*	6610	
Median [IQR] Monthly cases	484.5 [447.5, 540.5]	487.5 [463, 510]	506.5 [485, 524.5]	492 [479. 508]	503 [496.5, 530]	521.5 [504, 539]	549.5 [513.5, 581.5]	
Age								
Median [IQR]	60 [40, 76]	60 [41, 76]	60 [40, 76]	59 [40, 76]	59 [41, 75]	59 [40, 75]	59 [39, 75]	*p* = 0.11†
Gender								
Male (n) (%)	3222 (55.2%)	3194 (54.45)	3355 (55.0%)	3378 (57.2%)	3454 (55.9%)	2677 (54.2%)	3675 (55.6%)	*p* = 0.191•
Female (n) (%)	2615 (44.8%)	2675 (45.6%)	2743 (45.0%)	2527 (42.8%)	2721 (44.1%)	2258 (45.8%)	2929 (44.4%)	
Diabetes type								
Type 1(n) (%)	3125 (53.5%)	2962 (50.4%)	3026 (49.6%)	2737 (46.3%)	2872 (46.5%)	2264 (45.9%)	2906 (44.0%)	*p* < 0.001•
Type 2(n) (%)	2040 (34.9%)	2171 (37.0%)	2291 (37.6%)	2334 (39.5%)	2528 (40.9%)	1984 (40.2%)	2779 (42.0%)	
Unspec.(n) (%)	676 (11.6%)	740 (12.6%)	785 (12.9%)	840 (14.2%)	779 (12.6%)	690 (14.0%)	925 (14.0%)	
Emergency type								
Hyperglycemia (n) (%)	1452 (24.9%)	1488 (25.3%)	1616 (26.5%)	1722 (29.1%)	1966 (31.8%)	1701 (34.4%)	2455 (37.1%)	*p* < 0.001•
Hypoglycemia(n) (%)	4389 (75.1%)	4385 (74.7%)	4486 (73.5%)	4189 (70.9%)	4213 (68.2%)	3237 (65.6%)	4155 (62.9%)	

*•*Chi^2^ †KW test

*annual rate missing 4 months of data: September, October, November, December

### Overall model

**Fig. 2:**
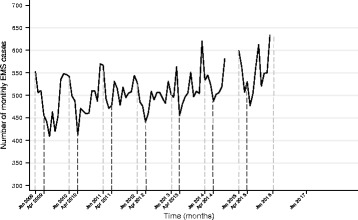
Observed monthly caseload of EMS attended cases for diabetic emergencies. Reference lines demonstrate seasonality, with peaks apparent around December/January, and troughs apparent around April/May

**Fig. 3:**
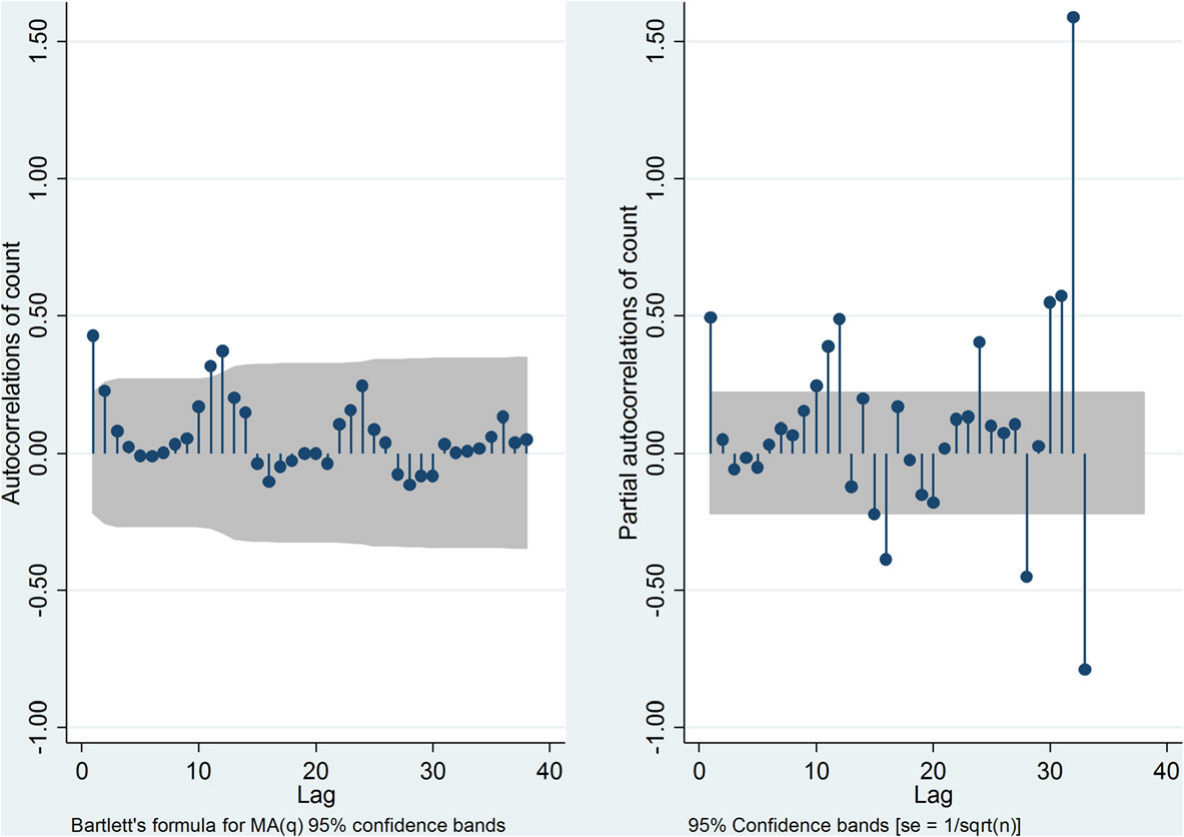
Autocorrelation plot and Partial autocorrelation plots for overall caseload of diabetic emergencies

**Fig. 4:**
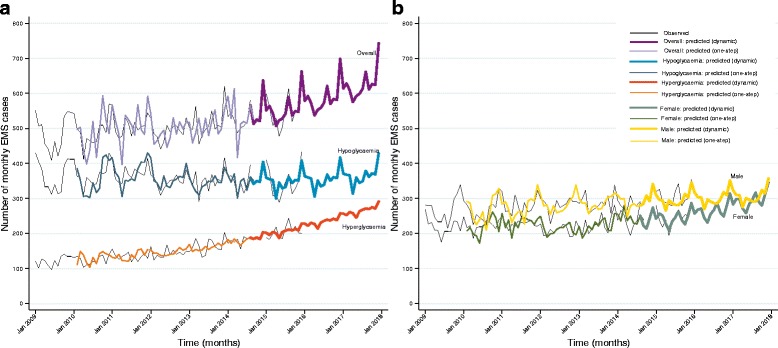
**a**: Time series plot of EMS attendance for diabetic emergencies (combined, hypoglycemia and hyperglycemia): observed, one-month forecast and dynamic forecast. **b**: Time series plot of EMS attendance for diabetic emergencies females and males): observed, one-month forecast and dynamic forecast

The monthly caseload of prehospital diabetic emergencies (Fig. 2) showed possible seasonality, with peaks apparent around December-January and troughs around April-May, as well as an overall increasing trend, suggesting a differencing term be incorporated in the model. The partial autocorrelation plot showed spikes at approximately month 12 and month 24 and the autocorrelation plot demonstrated an initial significant spike with a rapid decline and a peak at 12 months, both indicating the inclusion of a seasonality term (Fig. 3). The modelling process (Table 2) found the SARIMA (0,1,0) (0,1,0,12) term provided the best fit for the overall case counts, generating a MAE of 23.0, a MSE of 758.4 and a MAPE of 4.2% for the validation dataset. The predicted values of the final model (Fig. 4a, heavy purple line) demonstrate the increasing trend seen in the earlier data and predict a monthly caseload of 743 by the end of 2017, representing a 17% increase in monthly caseload from December 2015.

**Table 2:** Forecast errors for the various SARIMA models

Model	MAE	MSE	MAPE
Overall			
**(0,1,0,12)**	**22.96**	**758.39**	**4.23%**
(0,1,1,12)	24.88	870.29	4.63%
(1,1,0,12)	25.94	942.60	4.79%
(1,1,1,12)	24.54	862.56	4.56%
(0,1,2,12)	24.33	849.05	4.52%
(2,1,0,12)	23.79	794.36	4.41%
(2,1,1,12)	26.31	866.99	4.81%
(1,1,2,12)	26.09	909.21	4.81%
Hypoglycaemia			
(0,1,0,12)	38.12	2026.34	11.58%
**(0,1,1,12)**	**25.50**	**941.22**	**7.57%**
(1,1,0,12)	26.85	1055.83	8.04%
(1,1,1,12)	26.63	951.10	7.75%
(0,1,2,12)	27.34	994.96	7.90%
(2,1,0,12)	25.17	906.32	7.35%
(2,1,1,12)	27.84	1037.25	7.90%
(1,1,2,12)	27.73	1063.02	8.06%
Hyperglycaemia			
(0,1,0,12)	31.26	1412.35	14.74%
**(0,1,1,12)**	**12.94**	**254.31**	**6.26%**
(1,1,0,12)	15.49	321.36	7.43%
(1,1,1,12)	13.22	269.20	6.40%
(0,1,2,12)	13.38	281.07	6.49%
(2,1,0,12)	15.35	337.68	7.41%
(2,1,1,12)	15.24	348.73	7.41%
(1,1,2,12)	13.78	300.21	6.67%
Female			
(0,1,0,12)	26.21	918.92	10.81%
(0,1,1,12)	20.68	536.97	8.64%
(1,1,0,12)	22.40	632.35	9.31%
(1,1,1,12)	21.45	582.90	9.04%
(0,1,2,12)	20.76	547.93	8.75%
**(2,1,0,12)**	**14.78**	**360.32**	**6.12%**
(2,1,1,12)	17.93	414.51	7.47%
(1,1,2,12)	19.72	482.18	8.25%
Male			
(0,1,0,12)	24.97	872.20	7.97%
(0,1,1,12)	20.12	590.74	6.38%
(1,1,0,12)	22.34	746.52	7.03%
**(1,1,1,12)**	**18.85**	**618.37**	**5.99%**
(0,1,2,12)	18.97	634.50	6.03%
(2,1,0,12)	18.78	437.23	6.11%
(2,1,1,12)	20.70	605.84	6.56%
(1,1,2,12)	20.54	706.99	6.51%

MAE (Mean Absolute Error), MSE (Mean Square Error), MAPE (Mean Absolute Percentage Error)

Bold text indicates chosen model

### Hypoglycemia

The modelling process found the SARIMA (0,1,1) (0,1,1,12) term provided the best fit for cases of hypoglycemia, generating a MAE of 25.5, a MSE of 941.2 and a MAPE of 7.6% for the validation dataset. The predicted values of the final model (Fig. 4a, heavy blue line) suggest a stable trend of EMS-attended hypoglycemia cases in the short-term.

### Hyperglycemia

The modelling process found the SARIMA (0,1,1) (0,1,1,12) provided the best fit for cases of hyperglycemia, generating a MAE of 12.9, a MSE of 254.3 and a MAPE of 6.3% for the validation dataset. The predicted values of the final model (Fig. 4a, heavy orange line) suggest a rise in the number of EMS-attended cases of hyperglycemia, with a monthly caseload of 290 predicted by the end of 2017, representing a 45% increase from December 2015.

### Female

The modelling process found the SARIMA (2,1,0) (2,1,0,12) provided the best fit for female cases generating a MAE of 14.8%, a MSE of 360.3 and a MAPE of 6.1%. The predicted values of the final model (Fig. 4b, green line) suggest a rise in caseload of 24% between December 2015 and December 2017.

### Male

The modelling process found the SARIMA (1,1,1) (1,1,1,12) provided the best fit for male cases of diabetic emergencies generating a MAE of 18.9%, a MSE of 618.4 and a MAPE of 6.0%. The predicted values of the final model (Fig. 4b, golden line) suggest a very small rise in caseload of less than 1% between December 2015 and December 2017.

### Comparison of forecast horizon

The SARIMA (0,1,0,12) model was used to generate 1-month, 3-month and 12-month forecasts (Table 3). The shorter forecast horizon (1 month) demonstrated greatest accuracy (MAPE of 7.1%), and the 12-month projection demonstrated slightly greater accuracy than the 3-month projection, with a MAPE of 8.6% and 9.0%, respectively.

**Table 3:** 1 month, 3 month and 12 month forecasts for overall caseload SARIMA (0,1,0,12)

Model	MAE	MSE	MAPE
SARIMA (0,1,0,12)			
12 month projection	44.75	2195.23	8.57%
3 month projection	46.19	3173.26	9.00%
1 month projection	39.25	2031.35	7.14%

MAE (Mean Absolute Error), MSE (Mean Square Error), MAPE (Mean Absolute Percentage Error)

Generated using dynamic forecasting

### Comparison of time series models

When compared to other types of time series models, SARIMA (0,1,0,12) (one-step) generated a MAPE of 7.3%, out-performing b) SARIMA+ time trend (MAPE = 9.1%), d) exponential smoothing (MAPE = 8.8%) and e) linear time trend + seasonality (MAPE = 7.6%), and was comparable to c) ARIMA + seasonality (MAPE = 7.2%) (Table 4).

**Table 4:** Comparisons across models

	Model	MAE	MSE	MAPE
a)	SARIMA	37.75	1883.29	7.32%
b)	SARIMA + time trend	49.00	2792.45	9.11%
c)	ARIMA + seasonality	38.63	1581.40	7.23%
d)	Exponential smoothing	47.25	2384.75	8.78%
e)	Linear time trend + seasonality	43.22	2964.31	7.57%

MAE (Mean Absolute Error), MSE (Mean Square Error), MAPE (Mean Absolute Percentage Error)

Please note that measures of prediction accuracy across-model comparisons were generated using one-step forecasting

## Discussion

Utilization of prehospital EMS for acute diabetic emergencies is increasing with increased prevalence of diabetes. After separate analysis of the type of emergency, the majority of this increase appears to be due to cases of hyperglycemia. The SARIMA modelling process was able to model the monthly incidence of prehospital diabetic emergencies with good accuracy and predicts an increased caseload in the short term.

The temporal pattern of EMS demand for diabetic emergencies exhibited a long term rising trend with a seasonal component, where peaks were apparent around December-January (summer months) and troughs around April-May (autumn months). This is consistent with documented seasonal fluctuation in HbA1c levels showing lower levels in the summer months [23, 24]. Lower HbA1c levels are associated with an increased risk of severe hypoglycemia in people with type 1 [25] and type 2 diabetes [26]. Seasonal fluctuation has also been reported in hospital attendance and admission for hyperglycemia in people with type 2 diabetes [20]. The recognition of these season-related patterns may assist short-term organizational resource allocation, such as the increased service provision in the peak months.

While knowledge of seasonal fluctuations are useful for short-term service provision and rostering, the underlying increase of EMS use for diabetic emergencies throughout the study period demonstrates a need to address service provision longer-term. Cases of hypoglycemia and people with type 1 diabetes accounted for the majority of attendances, however, their relative proportions declined, with greater increases in attendances to cases of hyperglycemia and people with type 2 diabetes. This shifting demographic in demand for prehospital EMS has significant implications for the system in light of the types of patients who require transport to hospital. Local transport rates for people presenting with hyperglycemia are approximately 90%, much higher than those presenting with hypoglycemia (40%) [5]. Given transport to hospital consumes EMS resources for longer periods of time, and the types of patients who generally require hospital transport is increasing, consideration to long-term resource planning and sustainable alternatives to hospital transport are warranted. The forecast increases in EMS-attended diabetic emergencies highlights the importance of increasing routine detection of diabetes at the primary-care level and before emergency intervention is required as well as improved community-based management of diabetes, to avert some of the future caseload.

The International Diabetes Federation [27] reports that for the Western Pacific region (which includes Australia), the number of people with diabetes is estimated to increase from 153 million in 2015 to 215 million in 2040, representing a 1.6% annual increase in community prevalence of diabetes over the time period. Our model predicts a monthly caseload of 743 by the end of 2017, representing a 17% increase from December 2015, and an annual increase of approximately 8%, which far outweighs the predicted increase in community diabetes prevalence. Furthermore, between 2013 and 2015, the Australian National Diabetes Services Scheme register reported annual increases of prevalence of people with type 1 and type 2 diabetes of 0.6% and 4.4%, respectively. Over this same time period, there was an increase in EMS attendances for diabetic emergencies to people with type 1 and type 2 diabetes, suggesting the increases in EMS caseload may be partially explained by the increasing prevalence of diabetes, with the future predicted health burden likely to extend to prehospital EMS.

As predicted, using the 1-month forecast horizon resulted in greater accuracy than the 3-month and 12-month forecast horizon, however, the 12-month horizon performed better than the 3-month horizon. This could be explained by the inclusion of the 12-month seasonality term to the SARIMA models. Across model comparison showed that the selected SARIMA model resulted in a lower MAPE than SARIMA+ time trend, d) exponential smoothing and linear time trend + seasonality and a MAPE comparable to ARIMA + seasonality. While both SARIMA and ARIMA + seasonality are conceptually similar, SARIMA was selected due to simplicity in formulation and execution. It should be highlighted, however, that this is not a formal comparison of various time series models but rather a selection of the most appropriate model for the dataset.

The SARIMA (0,1,0) (0,1,0,12) provided the best fit for the overall caseload, generating a MAPE of 4.2%. Given the lack of published health forecast models in the prehospital field, and the variability of modelling approaches and methods of evaluation, comparison of our model’s performance is difficult. The MAE, MSE and MAPE were used in this study, however various other indices, such as route mean squared error (RMSE) [11] or goodness of fit criterion [9] have been used to evaluate performance of SARIMA models of emergency department caseloads. One study [13] developed an ARIMA model for forecast of daily rates of attendance at an emergency department and found a seasonal ARIMA (0,1,1)(1,0,1) yielded a MAPE of 4.8%, comparable to our results.

The use of time series modelling in health care to predict future events is increasing. Although there are many methods of time series prediction modelling, the ARIMA/SARIMA method has some distinct advantages. A study [28] comparing simple regression methods with ARIMA methods to predict acute hospital presentations argued that in the acute hospital environment, where trends can change suddenly and unpredictably, simple regression methods were not as effective forecasting tools as ARIMA, which can accommodate for autocorrelated data. In disease surveillance, ARIMA modelling was found to be favorable when compared to Bayesian processes to predict weekly incidence of Dengue fever [18]. Prediction models were based on weekly data from 2001–2006, and validated models on data between 2007 and 2008. Authors found that while the Bayesian K-H model provided marginally better prediction performance, this was outweighed by the relative ease of execution of ARIMA modelling.

The VACIS dataset captures all prehospital cases in the state of Victoria, Australia. The study is further strengthened by the prolonged timeframe of the data capture period (84 months), considered to be in the range of required observations for optimization (*n* = 50–100) [16]. Limitations of this study include the four months of missing data (Sept-Dec 2014), which impacted ability to generate 12-month forecasts for the validation period (2015) as well as the inherent limitations of forecast modelling. ARIMA models reflect and extend on past patterns. Thus, forecast accuracy is inversely related to the length of time of the forecast, whereby accuracy decreases with increasing length of forecast horizon as vulnerability to environmental or resourcing changes not accounted for in the model increases. Forecasting is also reliant on the reliability of the health data and robustness of the forecasting technique and accuracy is improved by updating the models as more data becomes available. A further limitation is that only cases listing hypoglycemia or hyperglycemia in the primary assessment were included, possibly underestimating the magnitude of the issue (as cases listing hypoglycemia or hyperglycemia as a secondary assessment were not included). The generalizability of the findings to other regions of the world may also be impacted by the local health care model and the socioeconomic and cultural background of those regions.

## Conclusions

In conclusion, ARIMA time series models forecast future prehospital caseload of diabetic emergencies with high accuracy. The results of this study demonstrate the requirement of appropriate health services planning and resource provision into the future with increases in both diabetes prevalence and pressures on prehospital emergency medical services anticipated. Further research regarding the effects of specific variables such as community prevalence of diabetes and climate factors, possible precipitants of diabetic emergencies and potential preventative measures to ease demand are required.

## Abbreviations

EMS: Emergency medical services
ARIMA: Autoregressive integrated moving average
AV: Ambulance Victoria
IQR: Interquartile range
AR: Autoregressive
MA: Moving average
SARIMA: Seasonal autoregressive integrated moving average
ACF: Auto correlation function
PACF: Partial autocorrelation function
MAE: Mean absolute error
MSE: Mean square error
MAPE: Mean absolute percentage error
